# Coronary Sinus Diameter as a Potential Marker of Right Ventricle Impairment

**DOI:** 10.3390/ijerph19042217

**Published:** 2022-02-16

**Authors:** Rafał Młynarski, Agnieszka Młynarska

**Affiliations:** 1 Department of Electroradiology, School of Health Sciences, Medical University of Silesia, 40-635 Katowice, Poland; rmlynarski@sum.edu.pl; 2 Department of Electrocardiology, Upper Silesian Heart Centre, 40-635 Katowice, Poland; 3 Department of Gerontology and Geriatric Nursing, School of Health Sciences, Medical University of Silesia, 40-635 Katowice, Poland

**Keywords:** right ventricle impairment, coronary sinus, ejection fraction, computed tomography

## Abstract

The aim of this study was to assess the influence of the parameters of the coronary sinus (CS) on the parameters that describe the function of the right ventricle (RV), which were calculated using cardiac computed tomography. Methods: A CT scan of the heart was performed on 150 patients due to suspicion of coronary artery disease using a Siemens Somatom Force (2 × 192 × 0.6) and a syngo.via workstation. The “CT coronary” and in some cases the generic presets were used to measure the CS ostium in millimeters (mm). The functional measurements of right heart ventricles were examined using the “CT cardiac function” automatic function on a 256 × 256 matrix. Results: The average diameter of the CS ostium was 16.29 ± 4.37 mm. In the group with RV impairment, it was 16.56 ± 4.76, whereas in the group with normal values of the RV, it was 15.98 ± 3.88 mm, *p* = 0.4199. The average angle of the entrance of the CS into the right atrium was 107.25° ± 9.68°. In the group with an RV impairment, it was 105.91° ± 9.22°, while in the patients with normal values of the RV, it was 108.82° ± 10.04°; *p* = 0.0682. A multiple regression showed that end systolic volume (*p* = 0.0017) and stroke volume (*p* = 0.0144) are important predictors of the CS ostium. Conclusions: Some relationships were found between the CS and the selected parameters that describe the function of the RV. This may suggest a role for the CS as a right ventricular buffer, which could potentially be treated as a marker of an RV impairment.

## 1. Introduction

**Figure 1:**
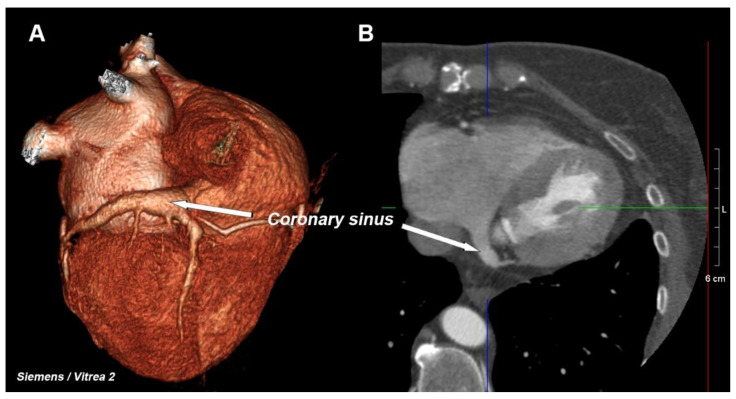
Example of CT image of the heart with coronary sinus visible. (**A**) 3D volume rendering. (**B**) 2D MPR multiplanar reformatted reconstruction.

The coronary venous system consists of several elements, the most important of which is the network of the veins of the heart (including the middle cardiac vein and the posterior, postero-lateral, lateral, antero-lateral, and anterior veins), from which blood flows into one large vessel that is called the great cardiac vein, which passes into the coronary sinus (CS)—Figure 1. This large vessel opens into the right atrium—this area is called the CS ostium [1,2]. The main clinical function of the coronary sinus is to drain most of the deoxygenated blood that leaves the heart muscle. Previous anatomical research has documented a large amount of anatomical variability of the coronary venous system [2,3]. This system, although it is often treated as an anatomical curiosity, has been the subject of many scientific studies for some time because of its use in electrophysiological procedures that have been widely used in the last two decades [4,5,6]. Most often, the CS is used as a retrograde pathway to one of the veins to ensure optimal left ventricular pacing. Together with right ventricular pacing, this is the widely accepted method for treating heart failure, which is called cardiac resynchronization therapy (CRT) [7]. Moreover, the available literature confirms the relationship of the coronary venous system with numerous heart pathologies such as heart failure and ischemic coronary artery disease [8,9].

Increased afterload is the major pathophysiological mechanism of right ventricle (RV) failure. Heart disease involving the right heart may primarily reduce RV contractility through decreased cardiac output or reduced RV preload, contributing to RV failure. The causes of right ventricular failure include myocardial ischemia/infarction, myocarditis, and most cardiomyopathies. Furthermore, pericardial disease can alter RV preload and ventricular interrelationship, while arrhythmias can exacerbate RV dysfunction [10,11,12]. In our opinion, coronary sinus diameter, due to its anatomical relationship with the right atrium and indirect connection with the right ventricle, may be an indicator of right ventricular function.

Most available papers have used only the hemodynamic parameters of the left heart. This may be due to the difficulty in assessing the RV using the available diagnostic methods [1,13] caused by the complicated shape of the right chamber, which is difficult to describe using mathematical formulas. Currently, the gold standard is the assessment of right ventricular function using magnetic resonance imaging, which is recommended by international experts [14,15]. In the last few years, rapid developments in computed tomography (CT) of the heart have enabled the evaluation of the right heart function parameters [13]. Therefore, the aim of this study was to assess the influence of the parameters of the CS ostium using a CT of the heart on the parameters that describe the function of the RV, which were calculated using cardiac CT. To authenticate our results, an additional aim was to assess the agreement of the obtained results between the researchers who took the measurements.

## 2. Materials and Methods

### 2.1. Study Design and Setting

One hundred and fifty consecutive patients (aged 63.6 ± 12.5; 75 women) were included in this prospective, single center study. The exclusion criteria were typical for a cardiac CT examination, such as severe renal failure (serum creatinine > 200 µmol/L or estimated glomerular filtration rate < 30 mL/min), major allergy to iodinated contrast agent, inability to give informed consent, and pregnant or lactating. CT scans of the heart in all of the patients were performed due to suspicion of coronary artery disease (CAD). The imaging was performed by a Siemens Somatom Force scanner (VB20_SP2) using 192 0.6 mm layers on a 512 × 512 matrix. Sixty ml of high-quality contrast agent was administered (Iomeron 400, Bracco, UK). A nitroglycerine dose was given according to the guidelines and, if needed, an intravenous B-blocker (metoprolol) was also administered to selected patients if it was not contraindicated. An automatic dose control program was used to fulfill the ALARA rules of being “as low as is reasonably achievable”.

The images were analyzed on the latest version of syngo.via (version VB30A_HF06). The “CT coronary” and, in some cases, the “MM (multi-modality)” presets were used to measure the CS ostium (largest diameter) in millimeters. The angle of the entrance of the CS into the right atrium was always measured between the long axis of the left ventricle in degrees and the half delineated straight through the ostium of the CS. Both calculations were performed on axial scans. The data were evaluated by two researchers experienced in coronary CT examinations (more than 20 years of experience).

**Figure 2:**
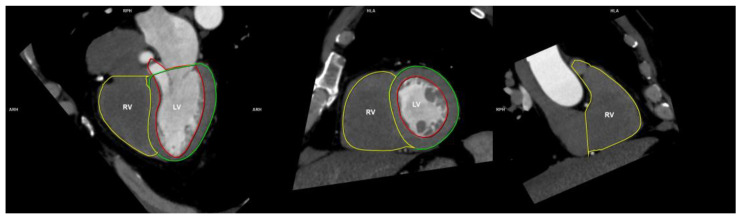
Scheme of the functional measurements of the RV. RV = right ventricle; LV = left ventricle.

The functional measurements of right heart ventricles were examined using the “CT cardiac function” automatic function on a 256 × 256 matrix. The scheme of the hemodynamic measurements of both ventricles is presented in Figure 2.

Finally, the patients were divided into two subgroups according to their RV ejection fraction (RV EF), which was calculated using cardiac CT: RV EF < 45% (impairment of RV) and RV EF ≥ 45%.

### 2.2. Ethical Considerations

The Medical University of Silesia ethics committee approved the study protocol (KNW/0022/KB/216/19). The study protocol complied with the version of the Helsinki Convention that was current at the time the study was designed. Due to the nature of the study, it was not necessary to obtain any additional written consent.

### 2.3. Statistical Analysis

The obtained data were statistically analyzed. The analysis of the quantitative variables was performed by calculating the mean ± standard deviation. The values of the quantitative variables in the two groups were compared using Student’s *t* test (when the variable had a normal distribution in these groups) or the Mann–Whitney test (otherwise). The normality of the distribution of the variables was determined using the Shapiro–Wilk test. Pearson correlation (r) analysis, which measures the strength of association between two variables, was also performed. Intra- and inter-observer agreement between CS measurements was evaluated in a subset of 30 patients (20%). The reproducibility of the measurements was measured using the Bland–Altman method and by calculating the inter-rater agreement coefficient kappa. A simple linear regression model was used to estimate the relationship between one independent variable and one dependent variable using a straight line. *p* values below 0.05 were interpreted as indicating significant dependencies. The analysis was conducted using MedCalc (Ostend, Belgium) and StatSoft Statistica 13 (Tulsa, OK, USA) software.

## 3. Results

Average body mass index (BMI) was 28.38 ± 5.16. The most prevalent risk factor was hypercholesterolaimia (99 out of 150 patients; 66%) and arterial hypertension (77 out of 150 patients; 51.3%). It is worth mentioning that diabetes was recognized in 33 out of 150 patients (22%). Thirty-four out of 150 patients ware active smokers or had been in the previous 5 years.

The first step of analysis was to evaluate the agreement between the two researchers who made the measurements. The inter-observer error for the CS diameter was 0.01 (95% CI 0.9053–0.9477) and the inter-rater agreement kappa was 0.92; for the angle of the entrance of the CS into the right atrium, the inter-observer error was 0.03 (95% CI 0.7262–0.8553) and the kappa was 0.79. The intra-observer error for the CS diameter was 0.01 (95% CI uth0.8994–0.9431) and the intra-rater agreement kappa was 0.92; for the angle of the entrance of the CS into the right atrium, the intra-observer error was 0.06 (95% CI 0.5917–0.8618) and the kappa was 0.72. The presented results are acceptable when measuring the CS.

The functional parameters of the RV, which were determined using a cardiac CT, are presented in Table 1.

The study included 16 (10,67%) patients with heart failure, including nine (6%) who had heart failure due to a reduced ejection fraction and seven (4.67%) who had heart failure with a preserved ejection fraction. The average diameter of the CS ostium was 16.29 ± 4.37 mm. In the group with an RV impairment, it was 16.56 ± 4.76; this was not statistically higher (*p* = 0.4199) compared to those patients with a normal-sized RV of 15.98 ± 3.88 mm. The average angle between CS and the right atrium was 107.25° ± 9.68°. In the group with an RV impairment, it was 105.91° ± 9.22°, while in patients with a normal-sized RV, it was 108.82° ± 10.04°; *p* = 0.0682.

**Figure 3:**
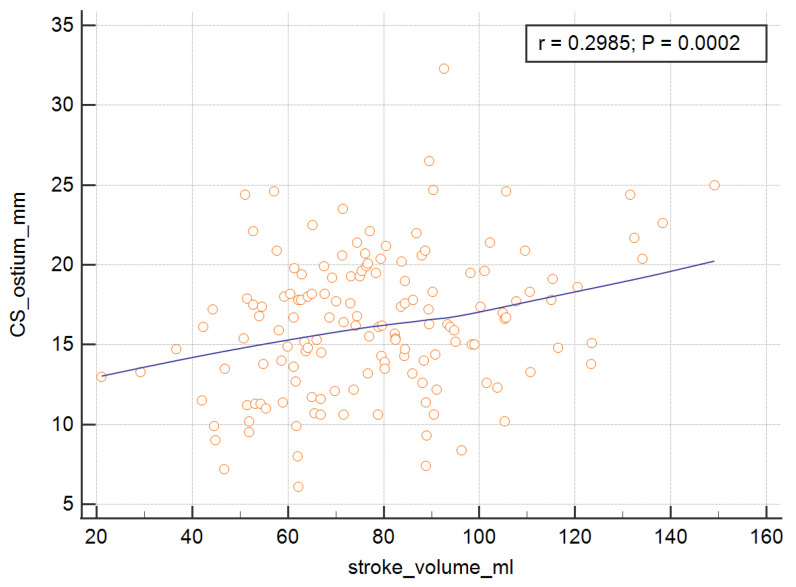
Correlation of the CS ostium with the stroke volume of the RV. CS = coronary sinus; RV = right ventricle.

**Figure 4:**
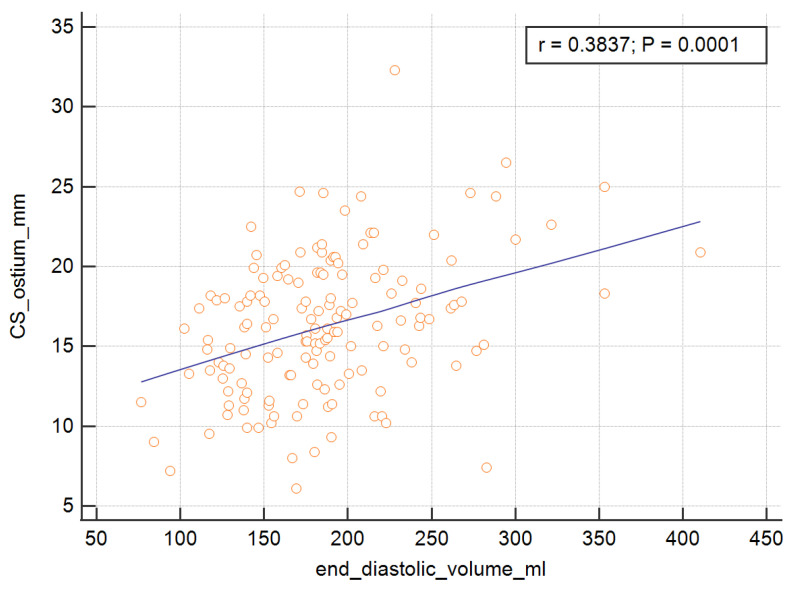
Correlation of the CS ostium with the end diastolic volume of the RV. CS = coronary sinus; RV = right ventricle.

**Figure 5:**
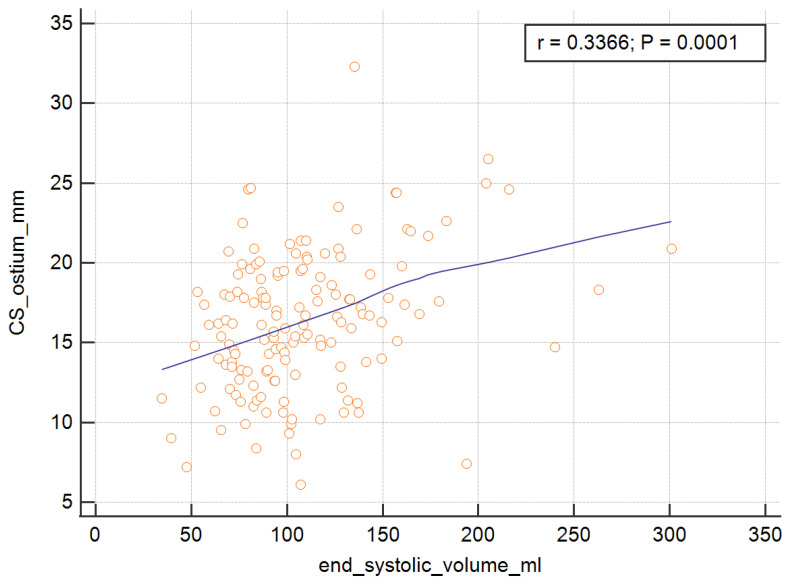
Correlation of the CS ostium with the end systolic volume of the RV. CS = coronary sinus; RV = right ventricle.

**Figure 6:**
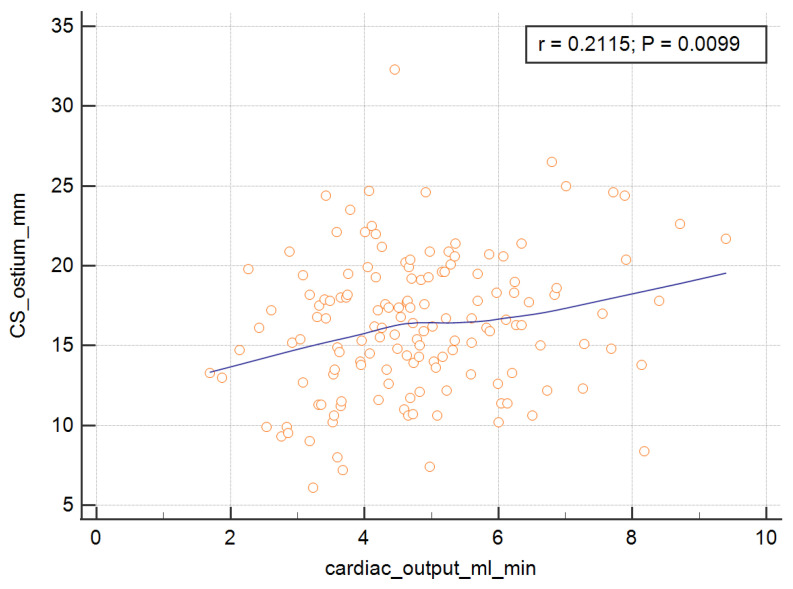
Correlation of the CS ostium with the cardiac output of the RV. CS = coronary sinus; RV = right ventricle.

In order to measure the ostium of the CS, four of the RV parameters, with the exception of the RV ejection fraction, were significant and are presented graphically in Figure 3, Figure 4, Figure 5 and Figure 6. The correlations of the CS ostium with the functional parameters of the RV according to the RV ejection fraction are presented in Table 2.

The correlations of the CS entrance angle into the right atrium ostium with the parameters that describe the function of the RV were not statistically significant at all. The exact data are presented in Table 3.

A few of the simple linear regression models that were used to estimate the relationship between the CS diameter and one dependent variable are presented in Table 4. The analysis showed that the SV, EDV, ESV, and CO were important predictors of the dependent variable.

## 4. Discussion

In this paper, we have documented that the cardiac venous system can be treated as a buffer of RV changes—when the four parameters of RV function increase, the width of the CS ostium also increases. Interestingly, there was no such relation between the angle of CS entry into the right atrium and the right ventricular ejection fraction. We can cautiously suggest that changes in the CS may indicate right ventricular failure—especially since it is much easier to calculate CS parameters than RV parameters. We presented a hypothesis that needs to be proven.

One of the rare studies that is similar to ours is the paper by Çakıcı et al., the aim of which was to evaluate CS dilatation in patients with heart failure and to demonstrate its relationship to the hemodynamic parameters of the RV [16]. The cited authors used a different imaging method than the one we used—echocardiography. The CS was analyzed in the apical four-chamber view. To evaluate an RV impairment, the RV myocardial performance index (RV MPI) was used (RV MPI > 0.55 was defined for impaired RV myocardial function). According to the results, the CS and RV MPI values were significantly higher in both the patients with ischemic and idiopathic DCM than in the healthy controls. In our research, in the group with an RV impairment, the CS ostium was not statistically larger than the one with normal RV function (16.56 ± 4.76 vs. 15.98 ± 3.88 mm, *p* = 0.4199). The cited Turkish authors predicting heart failure patients with an impaired RV function found a cut-off value for the diameter of the CS of 7.35. These authors concluded that the CS diameter can be used as a novel echocardiographic marker for RV impairment in patients with HF. While this is quite an interesting observation, we did not investigate it in the presented research.

The same group of authors under the supervision of Cetin investigated the relationship between the CS diameter and pulmonary artery systolic pressure in 100 of 155 patients who had been diagnosed with pulmonary hypertension and normal left ventricular systolic function on whom transthoracic echocardiography was performed [17]. An interesting method for the CS measurements in echocardiography was introduced during systole according to a formula in which the mean CS = (proximal CS + mid CS + distal CS)/3. The authors concluded that a dilated CS was associated with moderate to severe pulmonary hypertension, and that the RA area and PASP were independent predictors of the CS diameter.

In our opinion, although the results of the Turkish authors are extremely interesting, the quality of echocardiographic imaging for CS is often limited, especially when measuring small structures. This problem is rarely observed in a cardiac CT, the “gold standard” for visualizing anatomy, in which the quality of the venous system is usually very good. Unfortunately, unlike the echo exams, it exposes the patient to radiation and an iodine-based contrast agent to which some patients are allergic. It is difficult to perform a functional examination in CT, which is a strong point of echocardiography. All of the results suggest the necessity to assess the CS using all of the imaging methods, including echocardiography and CT. In 2012, Maffei et al. confirmed that despite its good agreement with magnetic resonance, CT is unlikely to become the preferred imaging investigative tool for the functional assessment of LV and RV because of the exposure to radiation and the need to administer a contrast material [18]. The situation has changed because both the radiation dose and the amount of contrast agent required have decreased significantly, and we believe that quite soon, we will have a new generation of CT for assessing the RV function together with the coronary venous system. As the technology of CT has advanced, taking these measurements has become easier. Of course, there are still some doubts about the methodology for measuring the RV using a cardiac CT, which we mentioned in the section on the limitations of the study; however, the pilot study that we performed showed that despite the doubts, there is 100% repeatability of the results of the parameters that describe the RV function, which encouraged us to present our results.

What is the clinical usefulness of the presented results? In our opinion, the clinical implications are limited to a few potential situations. When there are available CT raw images of the heart (DICOM) performed earlier for other reasons, we can use these data to evaluate the relations between RV and the CS. Another clinical situation is when there is a need for RV function evaluation in magnetic resonance imaging and the patient is excluded due to metal elements inside the body; in this case, CT is a good alternative. In our CT lab, we implemented an analysis of the cardiac venous system as a potential marker of right ventricular failure. To the best of our knowledge, our publication is the first to analyze the relationship between the RV and the venous system of the heart in CT.

### Limitations of the Study

The presented regressions, although statistically significant, should be considered weak.

## 5. Conclusions

Some significant relationships were found between the CS and selected parameters that describe RV function. This may suggest a role of CS diameter as a potential marker of right ventricle impairment.

## Figures and Tables

**Table 1:** Average values of the RV measurements using a cardiac CT. RV = right ventricle; CT = computed tomography; mL = milliliters.

RV Parameter	Average Value ± SD
RV ejection fraction (%)	42.76 ± 8.49
RV stroke volume (mL)	78.12 ± 22.76
RV end-diastolic volume (mL)	186.42 ± 54.14
RV end-systolic volume (mL)	108.33 ± 41.7
Cardiac output (L/min)	4.81 ± 1.48

**Table 2:** Correlation of the CS ostium with the parameters that describe the RV function according to the RV ejection fraction. CS = coronary sinus; RV = right ventricle.

RV Parameter	RV EF < 45% (Impairment of the RV)		RV EF ≥ 45% (Normal Function of the RV)	
RV ejection fraction (%)	r = −0.0704	*p* = 0.5351	r = −0.0448	*p* = 0.7166
RV stroke volume (mL)	r = 0.3429	*p* = 0.0018	r = 0.3142	*p* = 0.0091
RV end-diastolic volume (mL)	r = 0.4131	*p* = 0.0001	r = 0.3145	*p* = 0.0090
RV end-systolic volume (mL)	r = 0.3752	*p* = 0.0006	r = 0.2968	*p* = 0.0140
Cardiac output (L/min)	r = 0.2506	*p* = 0.0250	r = 0.2266	*p* = 0.0632

**Table 3:** Correlation of the CS entrance angle into the right atrium ostium with the parameters that describe the RV function. CS = coronary sinus; RV = right ventricle.

RV Parameter	Correlations	
RV ejection fraction (%)	r = 0.1344	*p* = 0.1035
RV stroke volume (ml)	r = −0.0455	*p* = 0.5828
RV end-diastolic volume (ml)	r = −0.1357	*p* = 0.1002
RV end-systolic volume (ml)	r = −0.1519	*p* = 0.0653
Cardiac output (l/min)	r = 0.0161	*p* = 0.8460

**Table 4:** Simple linear regression models that were used to estimate the relationship between the CS diameter and one dependent variable.

	R2	Coefficient	Std. Error	95% CI	*p*
CO	0.04473	0.07150	0.02735	0.01746–0.1256	0.0099
EDV	0.1473	4.7499	0.9460	2.8803–6.6194	<0.0001
EF	0.0083	−0.1770	0.1600	−0.4933–0.1392	0.2704
ESV	0.1133	3.2084	0.7429	1.7401–4.6767	<0.0001
SV	0.08909	1.55344	0.4111	0.7410–2.3659	0.0002

## Data Availability

Data sharing not applicable.
